# High-Performance Hybrid Phototheranostics for NIR-IIb Fluorescence Imaging and NIR-II-Excitable Photothermal Therapy

**DOI:** 10.3390/pharmaceutics15082027

**Published:** 2023-07-27

**Authors:** Qi Wang, Xinmin Zhang, Youguang Tang, Yanwei Xiong, Xu Wang, Chunlai Li, Tangxin Xiao, Feng Lu, Mengze Xu

**Affiliations:** 1 State Key Laboratory for Organic Electronics and Information Displays & Institute of Advanced Materials (IAM), Nanjing University of Posts & Telecommunications, Nanjing 210023, China; 2 Department of Liver Surgery, Shanghai Institute of Transplantation, Shanghai Engineering Research Center of Transplantation and Immunology, Renji Hospital, School of Medicine, Shanghai Jiao Tong University, Shanghai 200127, China; 3 Jiangsu Key Laboratory of Advanced Catalytic Materials and Technology, School of Petrochemical Engineering, Changzhou University, Changzhou 213164, China; 4 Center for Cognition and Neuroergonomics, State Key Laboratory of Cognitive Neuroscience and Learning, Beijing Normal University, Zhuhai 519087, China

**Keywords:** phototheranostics, organic–inorganic hybrid nanoagents, NIR-II light responsive, photothermal therapy, NIR-IIb fluorescence imaging

## Abstract

Photothermal therapy operated in the second near-infrared (NIR-II, 1000–1700 nm) window and fluorescence imaging in the NIR-IIb (1500–1700 nm) region have become the most promising techniques in phototheranostics. Their combination enables simultaneous high-resolution optical imaging and deep-penetrating phototherapy, which is essential for high-performance phototheranostics. Herein, carboxyl-functionalized small organic photothermal molecules (Se-TC) and multi-layered NIR-IIb emissive rare-earth-doped nanoparticles (NaYF_4_:Yb,Er,Ce@NaYF_4_:Yb,Nd@NaYF_4_, RENP) were rationally designed and successfully synthesized. Then, high-performance hybrid phototheranostic nanoagents (Se-TC@RENP@F) were easily constructed through the coordination between Se-TC and RENP and followed by subsequent F127 encapsulation. The carboxyl groups of Se-TC can offer strong binding affinity towards rare-earth-doped nanoparticles, which help improving the stability of Se-TC@RENP@F. The multilayered structure of RENP largely enhance the NIR-IIb emission under 808 nm excitation. The obtained Se-TC@RENP@F exhibited high 1064 nm absorption (extinction coefficient: 24.7 L g^−1^ cm^−1^), large photothermal conversion efficiency (PCE, 36.9%), good NIR-IIb emission (peak: 1545 nm), as well as great photostability. Upon 1064 nm laser irradiation, high hyperthermia can be achieved to kill tumor cells efficiently. In addition, based on the excellent NIR-IIb emission of Se-TC@RENP@F, in vivo angiography and tumor detection can be realized. This work provides a distinguished paradigm for NIR-IIb-imaging-guided NIR-II photothermal therapy and establishes an artful strategy for high-performance phototheranostics.

## 1. Introduction

Phototheranostics, which converts absorbed light into diagnosis signals and therapeutic functions, allowing real-time optical imaging and in situ phototherapy, has been considered as one of the burgeoning fields for both fundamental research and clinical applications [1,2,3]. Phototheranostics offers prominent advantages in the aspects of sensitive real-time diagnosis and simultaneous phototherapy with high spatiotemporal accuracy. This technique has been confirmed as an outstanding methodology for cancer theranostics. Indeed, optical imaging provides real-time high-resolution imaging from organelles to organs and has been widely investigated for pathological assay, clinical diagnosis, and imaging-guided surgery [4,5,6]. Meanwhile, phototherapy, including photothermal therapy (PTT), photodynamic therapy (PDT), and photocatalytic therapy, offers wonderful controllability, high treatment specificity, and low damage for normal tissues [7,8,9,10,11]. Therefore, extensive efforts have been dedicated to constructing highly efficient platforms for tumor phototheranostics recently.

Among all the phototherapy methodologies, photothermal therapy (PTT), which adopts photothermal agents (PTAs) to convert absorbed photons into hyperthermia, is an attractive tumor treatment modality [12,13,14,15,16]. Numerous studies have demonstrated that photothermal therapy exhibits remarkable efficacy in cancer treatment [17,18,19]. Compared to the traditional treatments like radiotherapy and chemotherapy, PTT possesses the superiorities of non-invasiveness, remote controllability, minimal side effects, and insignificant drug resistance [20,21,22]. To improve the efficiency of PTT, tremendous efforts have been made on the construction of high-performance PTAs. For instance, a BODIPY-based PTA with ultra-high photothermal conversion efficiency (PCE, 88.3%) was designed by Peng and co-workers via incorporating a barrier-free rotor in the *meso*-position [23]. However, the excitation wavelengths of the current PTT systems are mostly within the range of 650–900 nm (traditional near-infrared window, NIR-I), which inevitably impair the treatment outcome of deep-seated tumors due to the limited tissue penetration depth and strong background tissue absorption [24,25,26,27]. It is widely known that the absorption and scattering of light in biological tissue decrease as the light wavelength increases, achieving deeper tissue penetration and higher maximum permissible exposure (MPE) [28,29,30,31]. For instance, the MPE for a 1064 nm laser was 1 W/cm^2^, which was much higher than for a 808 nm laser (0.33 W/cm^2^). The higher MPE and tissue penetration will lead to significantly improved treatment outcomes of PTT. As a result, the fabrication of second near-infrared (NIR-II, 1000–1700 nm) light-responsive PTAs has great potential to realize outstanding therapeutic effect and become the leading topic of PTT research. Note that a majority of NIR-II-responsive PTAs are inorganic and polymer nanoparticles at present [32,33,34,35], while small-molecule-based NIR-II PTAs would be more promising because of their clear chemical structures, synthetic reproducibility, high biocompatibility, and tunable functional groups [36,37,38,39]. However, the design of NIR-II-responsive small-molecule-based PTAs is rather difficult, and NIR-II-absorbing small molecules with high photothermal efficiency and photostability are very scarce.

For optical imaging, NIR-I fluorescence imaging (FLI) has been proven to be an effective tool for the monitoring of oncotherapy owing to its irreplaceable superiorities of non-invasiveness, rapid feedback, low cost, and high sensitivity [40,41,42]. Nevertheless, the auto-fluorescence and photon scattering from the tissue, and the limited penetration depth of photons (the key issues for FLI) in NIR-I FLI are still huge unmet standards for high-quality bioimaging. To overcome these limitations, NIR-II fluorescence emission has been adopted as a novel FLI technique for in vivo imaging with high spatio-temporal resolution and large signal-to-background ratio (SBR), owing to the low tissue auto-fluorescence, reduced photon scattering, and deep tissue penetration [43,44,45,46,47,48]. NIR-II fluorescence imaging was considered as an unprecedented technique for optical imaging, which enables admirable imaging quality in vivo, especially for deeply buried tissues. This technique has even been applied for imaging-guided liver cancer resection in humans [49]. Despite the fact numerous phototheranostic nanoplatforms have been successfully constructed in the past few years, their therapeutic effect and diagnostic accuracy (mostly using NIR-IIa’ (1000–1300 nm) FLI and NIR-IIa (1300–1400 nm) FLI remain inadequate. Notably, in comparison with NIR-IIa’ and NIR-IIa biowindows, better imaging performance and higher SBR can be achieved by using the photons in the NIR-IIb (1500–1700 nm) sub-window due to the ultra-low signal interference [50,51,52,53]. Due to the energy band theory, the synthesis of NIR-IIb emitting organic fluorophores is tricky. Pb-based quantum dots can provide strong emission in the NIR-IIb region, while their potential biotoxicity hindered their further application. Compared with other materials, rare-earth-based nanoparticles with superior biocompatibility and unique optical properties have emerged as efficient NIR-IIb imaging agents recently [54,55,56,57]. For instance, Er^3+^- and Tm^3+^-doped nanoparticles can emit light around 1530 nm and above 1600 nm with high efficiency [58]. Carboxyl, amino, and thiol group functionalized molecules can be easily conjugated with rare-earth-doped nanoparticles to fabricate multifunctional theranostic platforms. For instance, carboxyl-group-modified cyanine dye can offer strong binding affinity towards rare-earth-doped nanoparticles, even in whole blood, 10% fetal bovine serum, and cell culture medium [59]. Nevertheless, to our knowledge, these rare-earth nanoparticles have not been adopted to construct phototheranostic nanoagents with NIR-IIb emission and NIR-II-excitable PTT functions, which could provide both high diagnosis accuracy and therapeutic outcomes.

**Scheme 1:**
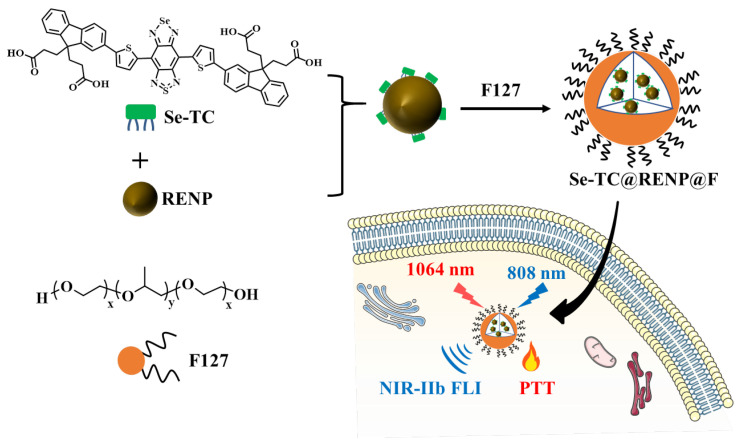
The chemical structure of Se-TC, and a graphical representation of the fabrication process of Se-TC@RENP@F as well as the application of Se-TC@RENP@F as phototheranostic nanoagents for 808 nm triggered NIR-IIb FLI and 1064 nm triggered PTT.

Herein, a carboxyl-functionalized small organic molecule (Se-TC) with a donor–acceptor–donor (D-A-D)-type core was first synthesized, whose maximum absorption peak was located at 920 nm in tetrahydrofuran (THF). Multilayered rare-earth-based nanoparticles (NaYF_4_:Yb,Er,Ce@NaYF_4_:Yb,Nd@NaYF_4_, RENP) with remarkable NIR-IIb emission were also skillfully designed and successfully prepared. Then, water-soluble organic–inorganic hybrid phototheranostic nanoagents (Se-TC@RENP@F) were easily constructed through the coordination between Se-TC and RENP and followed by subsequent F127 encapsulation (Scheme 1). The carboxyl groups could offer strong binding affinity of Se-TC to the surface of rare-earth-doped nanoparticles, which can improve the stability of Se-TC@RENP@F in aqueous solutions and blood. The Ce^3+^ ions in the core were doped to enhance the NIR-IIb emission of Er^3+^ ions by populating their ^4^I_13/2_ level through cross relaxation. The NaYF_4_:Yb,Nd shells were used to adjust the excitation wavelength of RENP from 980 nm to 808 nm due to the strong absorption of Nd^3+^ ions through ^4^I_9/2_ → ^4^F_5/2_ transition, which improves the imaging penetration depth and reduces the overheating effect. The resultant Se-TC@RENP@F exhibited excellent NIR-II absorption (extinction coefficient at 1064 nm: 24.7 L g^−1^ cm^−1^), bright NIR-IIb emission around 1545 nm, as well as great photostability. Upon 1064 nm laser irradiation, an outstanding photothermal effect with a high PCE value of 36.9% can be achieved, resulting in efficient tumor growth inhibition. Moreover, clear vascular imaging and accurate tumor diagnosis can be realized via NIR-IIb FLI. Overall, this work provides a proof-of-concept design strategy to develop NIR-IIb-imaging-guided, NIR-II-excitable photothermal nanoagents and hold great promise for the development of a variety of powerful phototheranostics.

## 2. Materials and Methods

### 2.1. Materials

Living/dead cell double staining kit, MTT assay kit, NIH-3T3 normal cells, HeLa cancer cells, and mice were purchased from Jiangsu KeyGEN BioTECH Corp., Ltd (Nanjing, China). F127 and other commercially available chemical reagents used for synthesizing Se-Tc were obtained from Energy-Chemical (Shanghai, China). Oleic acid, 1-octadecene, NH_4_F, and NaOH were obtained from Sigma-Aldrich LLC (Delaware, DE, USA). Sodium oleate was supplied by TCI (Tokyo, Japan). Rare-earth acetate hydrates (RE: Yb, Er, Ce, Nd, Y) were purchased from Alfa Aesar (Heysham, UK). In addition, all the chemicals used were not further purified. 

### 2.2. Synthesis of Se-TC

**Scheme 2:**
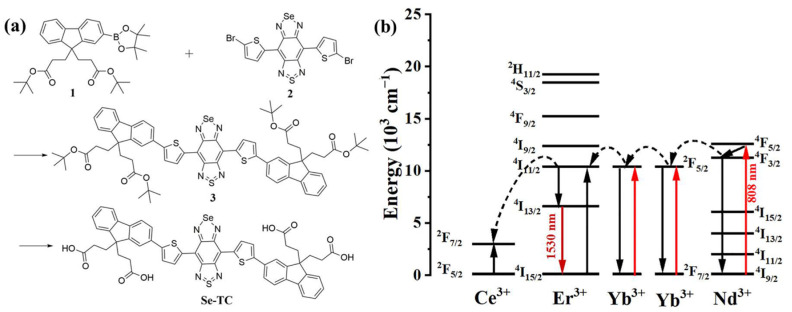
(**a**) Synthesis route of Se-TC. (**b**) Energy-level diagrams of RENPs upon 808 nm excitation.

The synthesis route of Se-TC is shown in Scheme 2a. Synthesis of compound **3**: Compound **1** [60] (154 mg, 0.281 mmol), compound **2** [61] (72 mg, 0.128 mmol), Pd(PPh_3_)_4_ (99 mg), K_2_CO_3_ (100 mg) were added in toluene (15 mL)/water (3 mL). The solution was stirred for 24 h at 95 °C under nitrogen, and the solvent was evaporated. Compound **3** could be obtained via column chromatography (84 mg, 52.6%). ^1^H NMR (400 MHz, CDCl_3_, δ): 9.11 (d, 2H), 7.85 (d, 2H), 7.80 (s, 2H), 7.74 (t, 4H), 7.64 (d, 2H), 7.43–7.36 (m, 6H), 2.47–2.42 (m, 8H), 1.43–1.33 (m, 8H), 1.31 (s, 36H). MALDI-TOF MS (*m*/*z*): [M]^+^ calcd. for C_68_H_70_N_4_O_8_S_3_Se, 1246.352; found, 1246.667. 

Synthesis of Se-TC: Compound **3** (80 mg, 0.064 mmol), TFA (3 mL) were added to DCM (15 mL). The solution was stirred for 12 h at room temperature. After extraction and washing, the solvent was evaporated, and a black solid was obtained, and directly used in the following step.

### 2.3. Synthesis of RENP

Synthesis of NaYF_4_:Yb,Er,Ce nanoparticles: 0.68 mmol Y(CH_3_CO_2_)_3_, 0.10 mmol Ce(CH_3_CO_2_)_3_, 0.20 mmol Yb(CH_3_CO_2_)_3_, and 0.02 mmol Er(CH_3_CO_2_)_3_ were mixed with 7 mL oleic acid and 15 mL 1-octadecene in a three-necked flask. The mixture was heated to 150 °C under nitrogen flow and maintained at this temperature for 40 min to dissolve the rare-earth acetates. Then, the solution was cooled to 50 °C. Next, 4.0 mmol NH_4_F and 2.5 mmol sodium oleate were dispersed in 10 mL methanol via sonication and added into the flask. After stirring at 50 °C for 40 min, the methanol was evaporated at 120 °C, and then the solution was heated to 290 °C for 1.5 h under nitrogen flow. The NaYF_4_:Yb,Er,Ce nanoparticles were precipitated with ethanol and washed with ethanol twice, and finally dispersed in 7 mL chloroform.

Synthesis of NaYF_4_:Yb,Er,Ce@NaYF_4_:Yb,Nd core–shell nanoparticles: 0.20 mmol Y(CH_3_CO_2_)_3_, 0.05 mmol Yb(CH_3_CO_2_)_3_, and 0.25 mmol Nd(CH_3_CO_2_)_3_ were dissolved in 7 mL oleic acid and 15 mL 1-octadecene at 150 °C under nitrogen protection. Then, the solution was cooled to 50 °C. An amount of 3.5 mL of the core solution was then added, along with 10 mL methanol containing 2.0 mmol NH_4_F and 1.25 mmol sodium oleate. The mixture was stirred at 50 °C for 40 min and then heated to 120 °C to remove the methanol. Subsequently, the solution was heated to 280 °C for 1.5 h under nitrogen flow. After cooling down to room temperature, the resulting NaYF_4_:Yb,Er,Ce@NaYF_4_:Yb,Nd nanoparticles were precipitated using ethanol, washed with ethanol twice, and re-dispersed in 5 mL chloroform.

Synthesis of NaYF_4_:Yb,Er,Ce@NaYF_4_:Yb,Nd@NaYF_4_ multilayered nanoparticles (RENP): 0.35 mmol Y(CH_3_CO_2_)_3_ was dissolved in 7 mL oleic acid and 15 mL 1-octadecene at 150 °C for 40 min under nitrogen flow. After cooling down to 50 °C, 5 mL of NaYF_4_:Yb,Er,Ce@NaYF_4_:Yb,Nd nanoparticles was added along with 10 mL methanol containing 1.4 mmol NH_4_F and 0.875 mmol sodium oleate. The mixture was stirred at 50 °C for 40 min and then heated to 120 °C to remove the methanol. Subsequently, the solution was heated to 280 °C under nitrogen protection for 1.5 h. After cooling down to room temperature, the RENPs were precipitated using ethanol, washed with ethanol twice, and finally re-dispersed in 7 mL chloroform.

### 2.4. Characterization

NMR spectra were recorded on a Bruker Ultra Shield Plus 400 MHz spectrometer (Bruker Corporation, Billerica, MA, USA) and referenced to tetramethylsilane (TMS) as the internal standard. Dynamic light scattering (DLS) studies were conducted using ALV/CSG-3 laser light scattering spectrometers (ALV-GmbH Corporation, Langen, Germany). Transmission electron microscopy (TEM) imaging was performed using a HT7700 transmission electron microscope (HITACHI, Tokyo, Japan). The absorption data were recorded using a Shimadzu UV-3600 ultraviolet–visible near-infrared spectrophotometer (Shimadzu Corporation, Kyoto, Japan), and photoluminescence spectra were measured using a Horiba Fluorolog 3 fluorescence spectrophotometer (Jobin Yvon Inc., Jasper, IN, USA) equipped with an 808 nm laser. Confocal laser scanning microscopy (CLSM) images were captured with ZEISS LSM 880 NLO microscope (Carl Zeiss AG, Oberkochen, Germany). In vivo NIR-II fluorescence images were acquired with a NIRvana 640 InGaAs FPA camera from Princeton Instruments (Acton, MA, USA) equipped with a 900 nm and a 1500 nm long pass filter (Thorlabs, Newton, MA, USA). The power density at the animal surface was adjusted to around 60 mW/cm^2^ for the excitation laser.

### 2.5. Preparation of Se-TC@RENP@F

First, TFH solution of Se-TC (400 μL, 1 mg/mL) was mixed with chloroform solution of RENP (200 μL, 10 mg/mL) into a glass bottle. After stirring, F127 (60 mg) was further added. Then, the mixed solution was rapidly added into water (10 mL) under ultrasound. After dislodging organic solvent, ultrafiltration, and washing, Se-TC@RENP@F solutions were obtained.

### 2.6. Photothermal Performance of Se-TC@RENP@F

Six concentrations of Se-TC@RENP@F solutions (0, 12.5, 25, 50, 100, and 200 µg/mL) were prepared and placed in centrifuge tubes. Each solution concentration was irradiated with a 1064 nm laser at a power density of 1.0 W/cm^2^, and the temperature of the Se-TC@RENP@F solution at different concentrations was measured using an FLIR E50 infrared thermal imager. Subsequently, Se-TC@RENP@F solution with a concentration of 200 µg/mL was irradiated with 1064 nm lasers at power densities of 1.0, 0.75, 0.5, and 0.25 W/cm^2^, and the temperature changes were measured and recorded. To calculate the PCE of Se-TC@RENP@F, Se-TC@RENP@F was irradiated by a 1064 nm laser for 5 min. After reaching a plateau temperature, the solution was naturally cooled to room temperature without NIR laser irradiation. And the temperature was recorded using an IR thermal camera. Deionized water of the same volume was used as control.

The PCE was calculated using equation [62]:

(1) $$\mathrm{PCE}=\frac{hs(T_{\max}-T_{\mathrm{surr}})-Q_{0}}{I(1-10^{-A_{\lambda }})}$$

*hs* can be calculated using the follow equation:

(2) $$hs=\frac{\sum m_{i}C_{P.i}}{\tau_{s}}$$

(3) $$\tau_{s}=\frac{t}{-\ln\theta }$$

(4) $$\theta =\frac{T-T_{\mathrm{surr}}}{T_{\max}-T_{\mathrm{surr}}}$$

(5) $$Q_{0}=hs(T_{\max}-T_{surr})$$

Here, *h* is the heat transfer coefficient, *s* represents the surface area of the sample container, *T*_max_ represents the maximum temperature of the sample solution after laser irradiation, *T*_surr_ is the ambient temperature, *Q*_0_ is the heat generated by solvent absorption of the laser, *I* represents the laser power, *A* is the absorbance of the solution at 1064 nm, *T* is the temperature during cooling, t represents the corresponding cooling time, *C* approximate to the specific heat capacity of water, and m is the mass of the sample solution.

### 2.7. Measurement of Extinction Coefficient

The absorption curves of Se-TC@RENP@F at different concentrations were measured using a UV-Vis-NIR spectrophotometer. The values at the absorption peak of 1064 nm were linearly fitted with their corresponding concentrations, and the slope was calculated using the corresponding formula to obtain the extinction coefficient.

### 2.8. MTT Assay

HeLa cells were grown in a 96-well plate. After the cells reached confluence, different concentrations of Se-TC@RENP@F (0, 20, 40, 60, 80, and 100 µg/mL) were added into the cells and incubated for 24 h. After washing, MTT solution was added, and the cells were further incubated for 5 h. After that, 120 µL of DMSO was added, and the absorbance at 490 nm was measured using an ELISA reader (Gene Company, Hong Kong, China). This process was used to test the dark toxicity of Se-TC@RENP@F. For phototoxicity testing, the cells were exposed to a 1064 nm laser (1 W/cm^2^) for 5 min during incubation with Se-TC@RENP@F.

### 2.9. Live and Dead Cell Assay

HeLa cells were cultured in 35 mm confocal dishes (4 × 10^5^ cells for each dish) for 24 h. Thereafter, HeLa cells were incubated with Se-TC@RENP@F at a concentration of 100 µg/mL for 5 h, and exposed to a 1064 nm (1 W/cm^2^) laser for 5 min. The cancer cells treated with different formulas were then stained with Calcein AM and PI for CLSM observations. 

### 2.10. In Vivo NIR-IIb FLI

Mice were obtained from KeyGEN BioTECH and used with the approval of the Animal Ethics Committee of Simcere BioTech Corp., Ltd. (Nanjing, China). Se-TC@RENP@F (150 μL, 1 mg/mL) were injected into HeLa tumor-bearing mice via the tail vein. The NIR-IIb images of the mice at different time points were captured using an NIR-II fluorescence imaging instrument equipped with a 1500 nm long pass filter (808 nm laser excitation).

## 3. Results and Discussion

### 3.1. Preparation and Characterization of Se-TC@RENP@F

**Figure 1:**
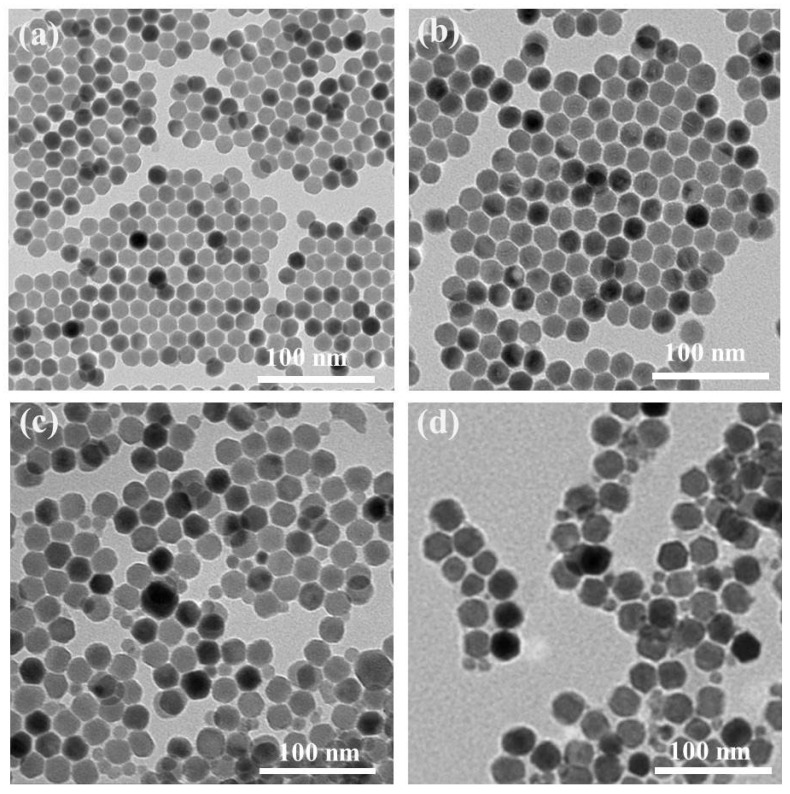
TEM images of (**a**) NaYF_4_:Yb,Er,Ce, (**b**) NaYF_4_:Yb,Er,Ce@NaYF_4_:Yb,Nd, (**c**) NaYF4:Yb,Er,Ce@NaYF4:Yb,Nd@NaYF4 (RENP), and (**d**) Se-TC@RENP@F.

**Figure 2:**
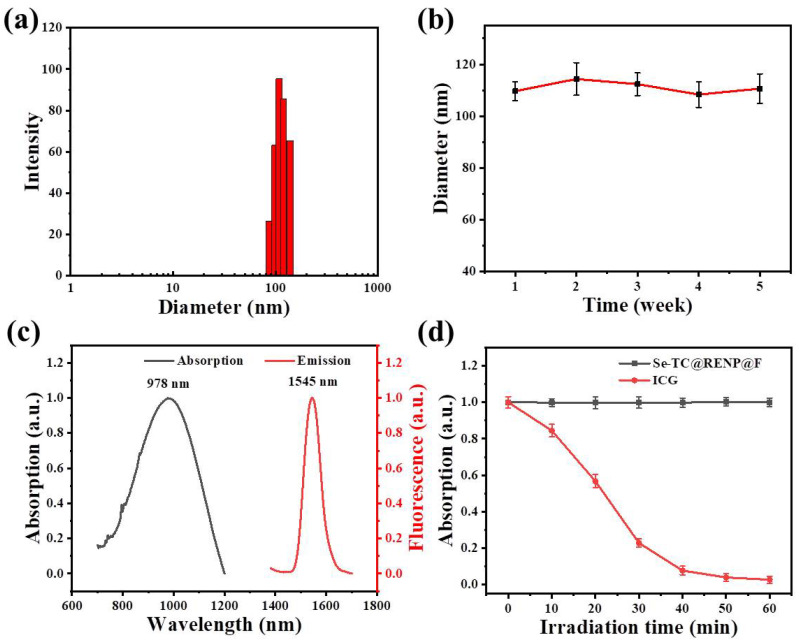
**Figure 2.** (**a**) Hydrodynamic size distribution (PDI: 0.319), (**b**) long-term stability, (**c**) normalized absorption and emission spectra, (**d**) photostability characterization of Se-TC@RENP@F in water.

Acceptors benzobisthiadiazole and thiadiazoloquinoxaline are usually used to design organic molecules with D-A-D structures. However, their maximum absorption peaks are only around 700 nm. Introducing stronger acceptors can enhance intramolecular charge transfer, resulting in the red-shifted absorption. Herein, the carboxyl-functionalized small organic molecule Se-TC was first prepared, in which [1,2,5]selenadiazolo[3,4-f]benzo[c][1,2,5]thiadiazole was used as an acceptor unit, and thiophene and fluorene were used as donor units (Scheme 2a and Scheme S1). The UV-vis-NIR survey displayed that Se-TC in THF possessed an evident absorption peak at 920 nm (Figure S3). The carboxyl groups of Se-TC can offer strong binding affinity towards rare-earth-doped nanoparticles, which help improve the stability of the obtained Se-TC@RENP@F in aqueous solution and blood [59]. A novel kind of NIR-IIb emitting fluorescent RENPs (NaYF_4_:Yb,Er,Ce@NaYF_4_:Yb,Nd@NaYF_4_) was then synthesized. The NaYF_4_:Yb,Er,Ce core was around 18.9 nm, as shown in Figure 1a. Ce^3+^ doping in the core can significantly populate the ^4^I_13/2_ level of Er^3+^, which leads to an improved down-conversion luminescence around 1550 nm [63]. The core–shell NaYF_4_:Yb,Er,Ce@NaYF_4_:Yb,Nd was then obtained as 21.6 nm nanoparticles, as shown in Figure 1b. Here, the Nd^3+^-doped sensitizing layer makes the nanoparticles excitable at around 800 nm [64]. Finally, the NaYF_4_ inert shell was coated to reduce the surface defects and the quenching effect from water molecules. Under 808 nm laser excitation, the Nd^3+^ ions will absorb photons and transfer the energy to Yb^3+^, and finally to the Er^3+^ in the core to emit light around 1530 nm (Scheme 2b). Quasi-spherical morphology of RENP (diameter: around 24.4 nm) was observed from the TEM image (Figure 1c). The FL spectrum showed that RENP exhibited evident NIR-IIb emission peak (1531 nm, Figure S4). To obtain a high-performance phototheranostic nanoagent Se-TC@RENP@F, RENPs in chloroform and Se-TC in THF were first mixed together. After stirring for 1 h, F127 was further added. Then, the mixed solution was rapidly added into water under ultrasound. After centrifugation and washing, Se-TC@RENP@F were obtained. As illustrated in the TEM image (Figure 1d), Se-TC@RENP@F exhibited a spherical morphology with a certain degree of aggregation, which is common for amphiphilic polymer-encapsulated nanoparticles [65,66]. The hydrodynamic diameter was determined to be about 110 nm by DLS, which was suitable for biomedical applications (Figure 2a). Notably, Se-TC@RENP@F displayed high stability (Figure 2b). In comparison with Se-TC in THF, the maximum absorption peak of Se-TC@RENP@F was red-shifted by about 58 nm to 978 nm (Figure 2c). Interestingly, the absorption of Se-TC@RENP@F could even extend to 1200 nm, matching well with the 1064 nm NIR-II excitation light. In addition, the small absorbance around 740 nm and 800 nm can be attributed to the absorption of Nd^3+^ ions in RENP. Moreover, the extinction coefficient of Se-TC@RENP@F at 1064 nm was 24.7 L g^−1^ cm^−1^, suggesting their good light absorption ability (Figure S5). Next, we analyzed the FL spectrum of Se-TC@RENP@F (Figure 2c). As anticipated, Se-TC@RENP@F emitted outstanding NIR-IIb fluorescence signals (peak: around 1545 nm), which were similar to those of the RENP. Moreover, the excellent photostability of Se-TC@RENP@F was also corroborated by continuous laser irradiation (Figure 2d), which was much better compared to the commercial NIR imaging agent indocyanine green (ICG).

### 3.2. Photothermal Performance of Se-TC@RENP@F

**Figure 3:**
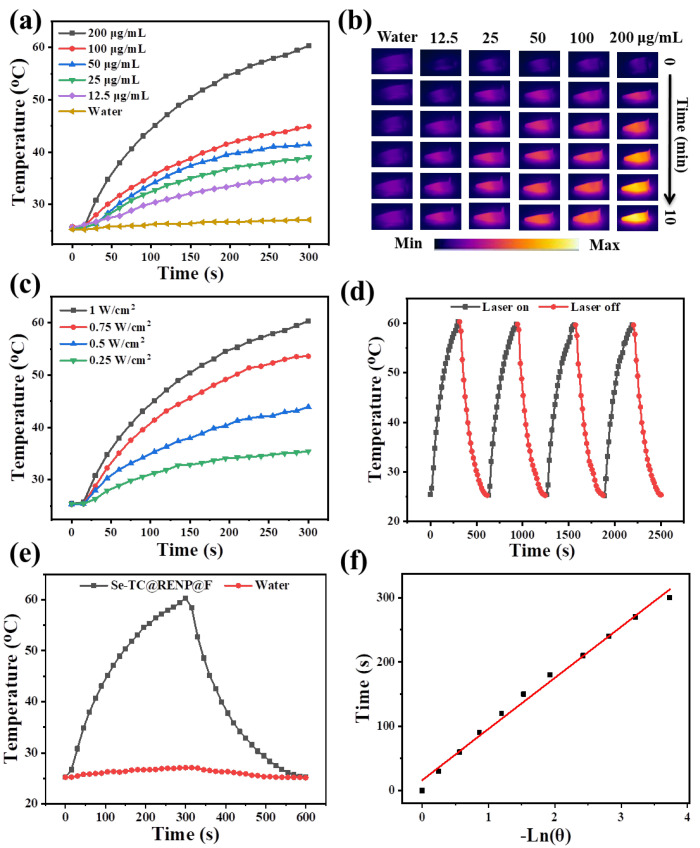
The evaluation of photothermal effects of Se-TC@RENP@F by changing the (**a**) Se-TC@RENP@F concentration (1064 nm laser illumination, 1 W/cm^2^) and (**c**) laser intensity (Se-TC@RENP@F: 200 μg/mL). (**b**) IR thermal images of Se-TC@RENP@F at different concentrations. The evaluation of the (**d**) photothermal stability of Se-TC@RENP@F. (**e**) The temperature variation curves of Se-TC@RENP@F and water under 1064 nm laser irradiation, followed by naturally cooling without the laser. (**f**) The linear cooling time versus −ln(θ) obtained from the cooling period of (**e**).

Subsequently, the NIR-II-excitable photothermal conversion capability of Se-TC@RENP@F was investigated. As expected, 5 min of illumination with a 1064 nm laser elevated the temperature of Se-TC@RENP@F (200 μg/mL) to 60.3 °C, yet the rise in the temperature of pure water was negligible (Figure 3a). Meanwhile, a higher temperature elevation could be achieved with the increase in concentration. Infrared (IR) thermal images of Se-TC@RENP@F at different concentrations were collected and used to demonstrate the corresponding temperature changes (Figure 3b). In addition, the photothermal effects of Se-TC@RENP@F under 1064 nm laser illumination with different laser intensities were studied (Figure 3c). The photothermal effects of Se-TC@RENP@F showed a laser-intensity-dependent manner; the higher power density of the laser led to a higher sample temperature. In addition, Se-TC@RENP@F preserved negligible temperature alteration after four cycles of heating/cooling, suggesting the excellent photothermal stability of Se-TC@RENP@F under the experimental condition (Figure 3d). Furthermore, the PCE of Se-TC@RENP@F at 1064 nm was calculated as 36.9% based on a cycle of heating/cooling (Figure 3e,f), which is better than many cyanine dyes and gold-nanorod-based photothermal agents [67,68,69].

### 3.3. Cytotoxicity Assay of Se-TC@RENP@F

**Figure 4:**
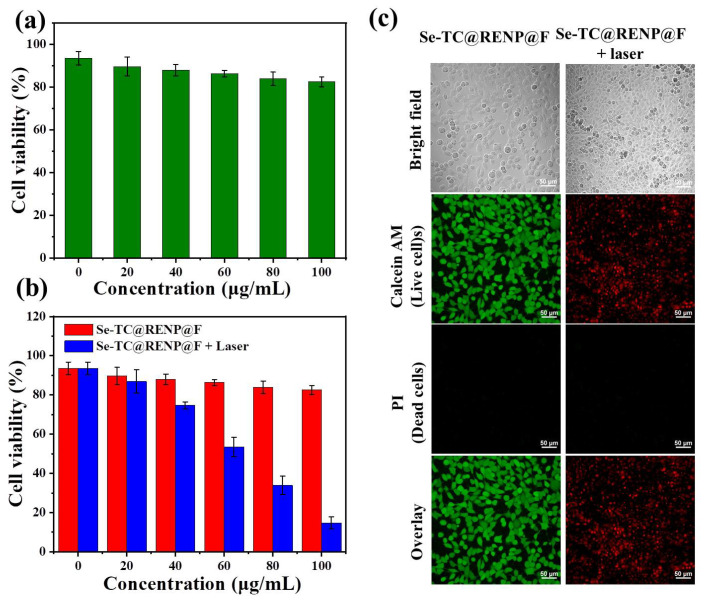
(**a**) Biocompatibility test of Se-TC@RENP@F by MTT assay. Treatment effect of Se-TC@RENP@F upon 1064 nm laser illumination assessed by (**b**) MTT and (**c**) live/dead cell staining assays.

The biosafety of Se-TC@RENP@F was investigated next. As illustrated in Figure 4a, Se-TC@RENP@F exhibited negligible cytotoxicity toward NIH-3T3 normal cells with the concentration even up to 100 μg/mL, indicating their good biosafety in vitro. In addition, after injection of Se-TC@RENP@F into healthy mice, insignificant weight loss was found, further suggesting the good biosafety of Se-TC@RENP@F in vivo (Figure S6). Then, we studied the in vitro photothermal ablation against HeLa cancer cells by Se-TC@RENP@F. From the results of the MTT assay (Figure 4b), insignificant toxicity of Se-TC@RENP@F was observed without 1064 nm laser illumination. Nevertheless, upon 1064 laser illumination, the viabilities of HeLa cells reduced obviously in a concentration-dependent manner because of the outstanding photothermal effect of Se-TC@RENP@F. The in vitro treatment effect of Se-TC@RENP@F was further confirmed by live/dead cell staining assay. Calcein AM and PI were used to stain live and dead cells, respectively. As presented in Figure 4c, cells incubated with Se-TC@RENP@F without laser treatment remained alive (broad green fluorescence), whereas cells in Se-TC@RENP@F + laser group were almost totally killed (intensive red fluorescence). These data demonstrated the biocompatibility and the photothermal therapeutic ability of Se-TC@RENP@F.

### 3.4. In Vivo NIR-IIb FLI

**Figure 5:**
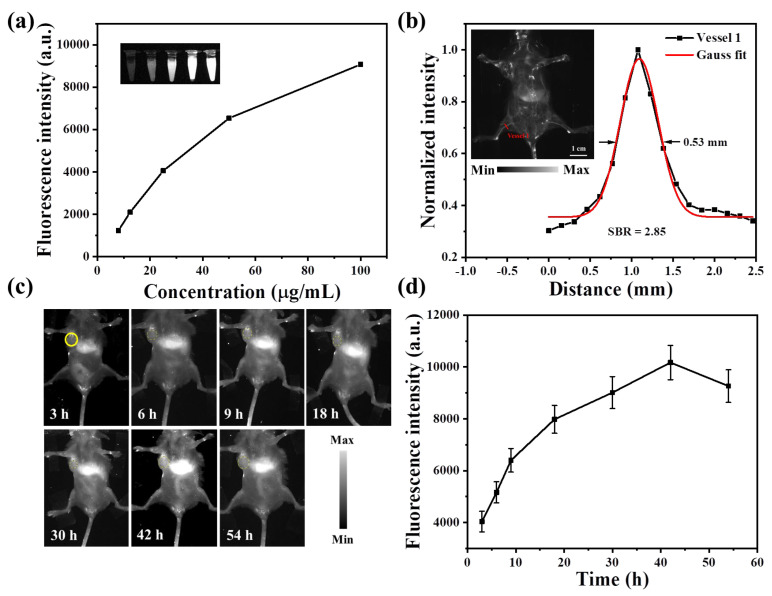
(**a**) NIR-IIb fluorescence intensity and NIR-IIb brightness of Se-TC@RENP@F at various concentrations. (**b**) The mensuration of full width at half maximum and SBR of the selected vessel. Inset: NIR-II angiography at 5 min post injection of Se-TC@RENP@F. (**c**) NIR-IIb FLI of mice at different time points based on Se-TC@RENP@F (the yellow circle represents the tumor area). (**d**) The tumor area’s NIR-IIb signal intensities at various time points.

Motivated by the excellent NIR-IIb fluorescence emission performances of Se-TC@RENP@F, the potential of Se-TC@RENP@F as NIR-IIb imaging reagents was then studied. The in vitro NIR-IIb fluorescence intensities of Se-TC@RENP@F at various concentrations were first examined (Figure 5a). As the concentration increased, both NIR-IIb fluorescence intensity and NIR-IIb brightness elevated. Then, the in vivo NIR-IIb FLI capacity of Se-TC@RENP@F was studied in xenograft HeLa-tumor-bearing mice. The vascular structure of mice could be observed upon intravenous administration of Se-TC@RENP@F at 5 min post injection. For example, the “full width at half maximum” and SBR of the selected vessel in hind limb were determined to be 0.53 mm and 2.85, respectively (Figure 5b). The tumor area’s NIR-IIb signal intensities at various time points were further measured by injection of Se-TC@RENP@F into other mice. As shown in Figure 5c,d, tolerable NIR-IIb signals could be found at 3 h post injection, and the intensities of NIR-IIb signal expanded by degrees with time. This observation indicated a passive targeting ability of Se-TC@RENP@F in tumors due to the enhanced permeability and retention (EPR) effect of the nanoparticles. Since nanoparticles were usually eliminated from the blood circulation by the reticulo-endothelial system [70], Se-TC@RENP@F also gradually accumulated in the liver and spleen. As a result, these deeply buried organs could be clearly visualized due to the high penetration of photons in the NIR-IIb region (Figure 5c). After 42 h post injection, the intensities of NIR-IIb signal declined step-wise. Hence, 42 h post injection was considered to be the maximum accumulation time at the tumor site for Se-TC@RENP@F in vivo. These results demonstrated that Se-TC@RENP@F could be used as good NIR-IIb imaging reagents.

## 4. Conclusions

By utilizing longer wavelengths (1500–1700 nm), NIR-IIb imaging offers deeper tissue penetration, reduced scattering, and eliminated autofluorescence, resulting in enhanced imaging resolution and sensitivity. In addition, photothermal therapy operated with NIR-II light, enhances therapeutic outcomes due to the deeper tissue penetration, reduced scattering, higher maximum permissible exposure, and minimized phototoxicity. As a result, fluorescence imaging in the NIR-IIb region and photothermal therapy in the NIR-II window have become the most heavily explored aspects in phototheranostics at present. And their integration has been considered as the one of the most promising approaches for imaging-guided phototherapy. In this work, we rationally designed and successfully developed high-performance hybrid phototheranostic Se-TC@RENP@F nanoagents, specifically for NIR-IIb FLI guided NIR-II-excitable PTT. The nanoagents were based on the novel carboxyl-functionalized small molecule Se-TC, rare-earth-based NIR-IIb fluorophores RENP, and the amphiphilic block copolymers F127. Compared with Se-TC in THF, the absorption peak of Se-TC@RENP@F red-shifted to 978 nm, and could even extend to 1200 nm with a high extinction coefficient in the NIR-II region (24.7 L g^−1^ cm^−1^ at 1064 nm). Upon 1064 nm laser irradiation, high hyperthermia with a large PCE value of 36.9% can be achieved, effectively inhibiting tumor cell growth. Moreover, clear vascular imaging and accurate tumor diagnosis can be materialized via in vivo NIR-IIb FLI of Se-TC@RENP@F. Overall, this study provides a proof-of-concept strategy to design nanoagents for cancer diagnosis and treatment and holds great promise for the development of various high-performance phototheranostic nanoplatforms.

## Data Availability

All data are contained within the article or supplementary material.

## Supplementary Materials

The following supporting information can be downloaded at: https://www.mdpi.com/article/10.3390/pharmaceutics15082027/s1, Scheme S1: Synthesis of Se-TC; Figure S1: ^1^H NMR spectrum of compound **3**; Figure S2: MALDI-TOF MS plot of compound **3**; Figure S3: Absorption spectrum of Se-TC in THF; Figure S4: NIR-II emission spectrum of RENP in chloroform; Figure S5: (a) Absorption curves of Se-TC@RENP@F at different concentrations. (b) Linear absorbance versus concentration obtained from (a); Figure S6: The body weight changes of healthy mice treated with PBS and Se-TC@RENP@F.
