# Supplementary material for: High-Performance Hybrid Phototheranostics for NIR-IIb Fluorescence Imaging and NIR-II-Excitable Photothermal Therapy
Source: Pharmaceutics. 2023 Jul 27;15(8):2027. doi: 10.3390/pharmaceutics15082027 (PMC10457990; doi:10.3390/pharmaceutics15082027)
Supplement: Supplementary file 1 [file pharmaceutics-15-02027-s001.zip › pharmaceutics-2486221-supplementary.pdf]

Supplementary Materials

High-Performance Hybrid Phototheranostics for NIR-IIb Fluorescence Imaging and NIR-II-Excitable Photothermal Therapy

Qi Wang [1], Xinmin Zhang [1], Youguang Tang [1], Yanwei Xiong [1], Xu Wang [1], Chunlai Li [2,*], Tangxin Xiao [3], Feng Lu [1,*] and Mengze Xu [4,*]

[1] State Key Laboratory for Organic Electronics and Information Displays & Institute of Advanced Materials (IAM), Nanjing University of Posts & Telecommunications, Nanjing 210023, China

[2] Department of Liver Surgery, Shanghai Institute of Transplantation, Shanghai Engineering Research Center of Transplantation and Immunology, Renji Hospital, School of Medicine, Shanghai Jiao Tong University, Shanghai 200127, China

[3] Jiangsu Key Laboratory of Advanced Catalytic Materials and Technology, School of Petrochemical Engineering, Changzhou University, Changzhou 213164, China

[4] Center for Cognition and Neuroergonomics, State Key Laboratory of Cognitive Neuroscience and Learning, Beijing Normal University, Zhuhai 519087, China

* Correspondence: chunlai.lee@gmail.com (C.L.); iamflu@njupt.edu.cn (F.L.); mengzexu@bnu.edu.cn (M.X.)

1. Synthesis of Se-TC

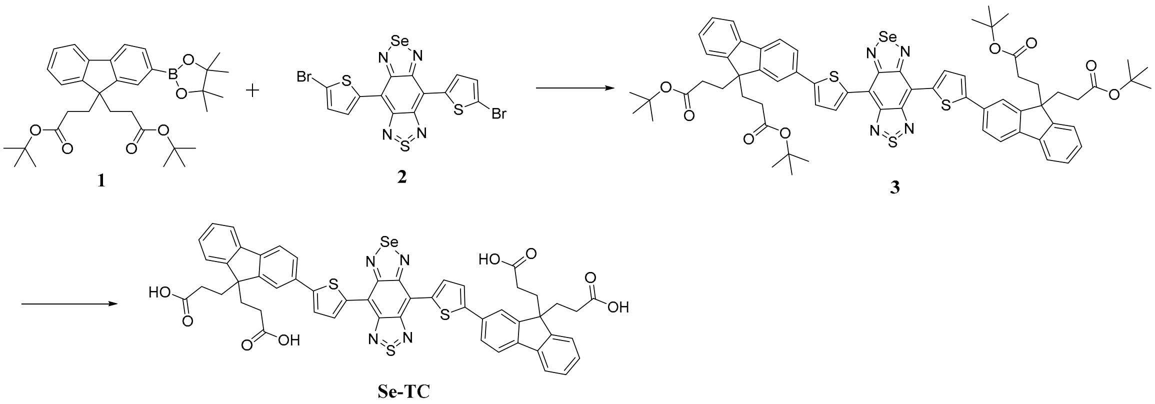

Scheme S1. Synthesis of Se-TC.

Synthesis of compound 3

Compound 1[S1] (154 mg, 0.281 mmol), compound 2[S2] (72 mg, 0.128 mmol), $Pd(PPh_3)_4$ (99 mg), K_2CO_3 (100 mg) were added in toluene (15 mL)/water (3 mL). The solution was stirred for 24 h at 95°C under nitrogen, and the solvent was evaporated. Compound 3 could be obtained via column chromatography (84 mg, 52.6%). ^{1}H NMR (400 MHz, $CDCl_3$, δ): 9.11 (d, 2H), 7.85 (d, 2H), 7.80 (s, 2H), 7.74 (t, 4H), 7.64 (d, 2H), 7.43-7.36 (m, 6H), 2.47-2.42 (m, 8H), 1.43-1.33 (m, 8H), 1.31 (s, 36H). MALDI-TOF MS (m/z): $[M]^+$ calcd. for $C_{68}H_{70}N_4O_8S_3Se$, 1246.352; found, 1246.667.

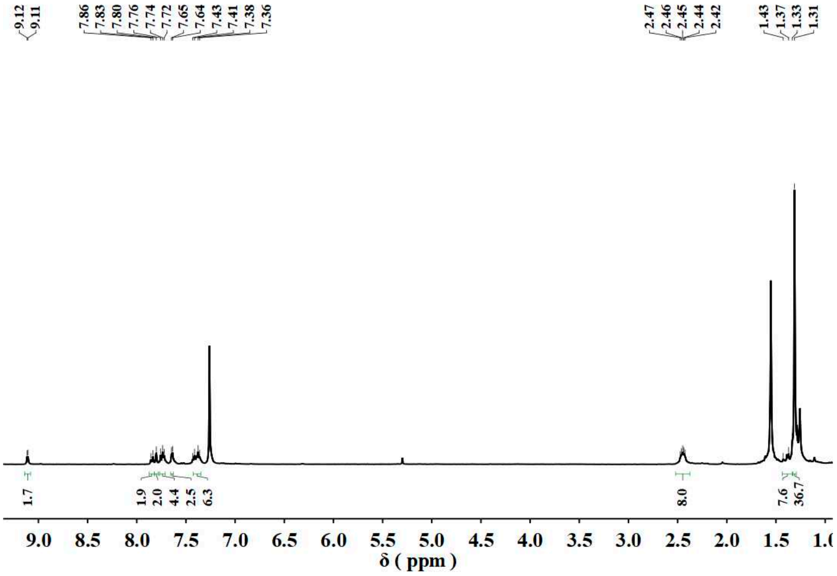

Figure S1. ^{1}H NMR spectrum of compound 3.

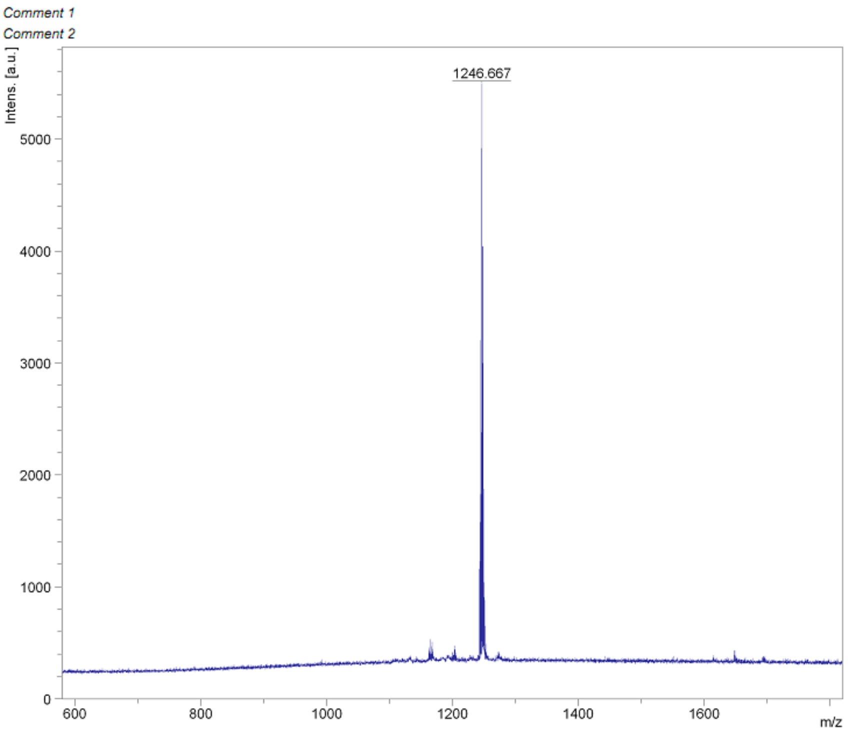

Figure S2. MALDI-TOF MS plot of compound 3.

Synthesis of Se-TC

Compound 3 (80 mg, 0.064 mmol), TFA (3 mL) were added in DCM (15 mL). The solution was stirred for 12 h at room temperature. After extraction and washing, the solvent was evaporated, and a black solid was obtained, and directly used in the following step.

2. *Absorption spectrum of Se-TC in THF*

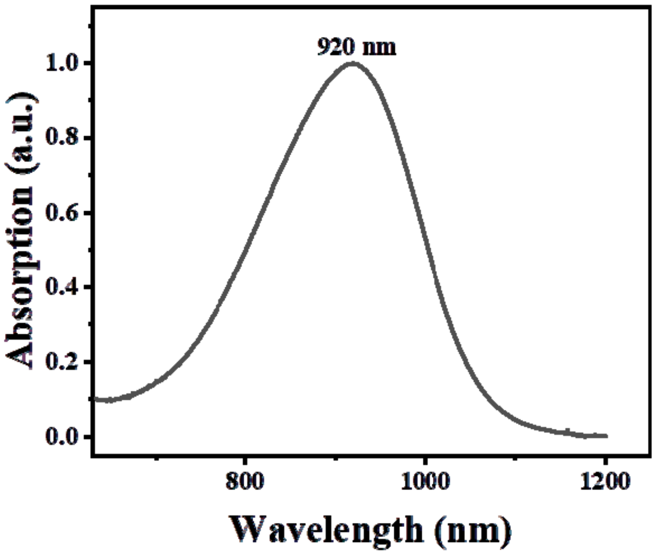

Figure S3. Absorption spectrum of Se-TC in THF.

3. *NIR-II emission spectrum of RENP in chloroform*

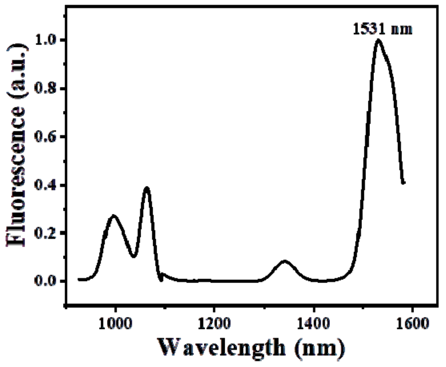

Figure S4. NIR-II emission spectrum of RENP in chloroform.

4. *Extinction coefficient of Se-TC@RENP@F*

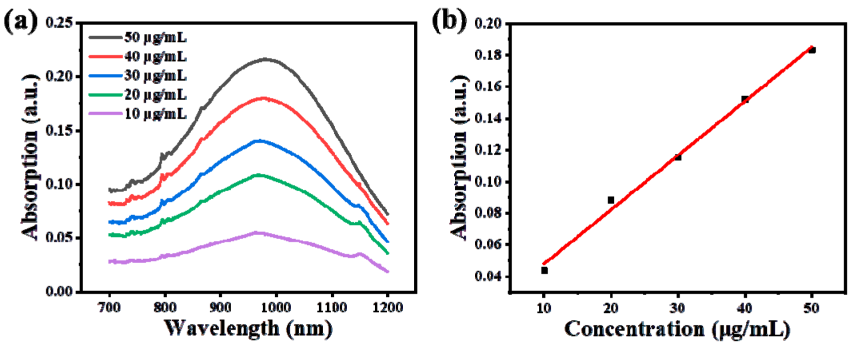

Figure S5. (a) Absorption curves of Se-TC@RENP@F at different concentrations. (b) Linear absorbance versus concentration obtained from (a).

5. *The biocompatibility of Se-TC@RENP@F in vivo*

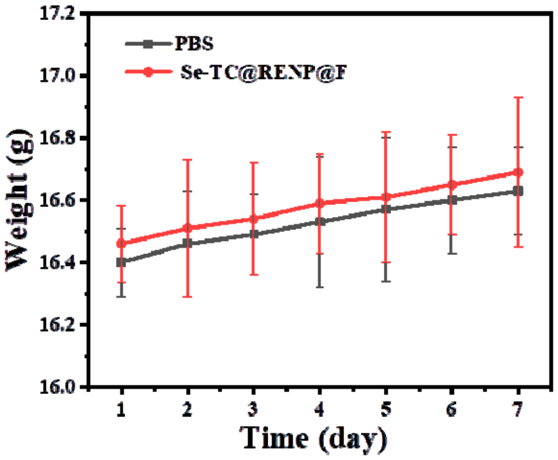

Figure S6. The body weight changes of healthy mice treated with PBS and Se-TC@RENP@F.

References:

[S1] X. Liu, R. Zhu, Y. Zhang, B. Liu, S. Ramakrishna, *Chem. Commun.* **2008**, 3789–3791, doi: 10.1039/B805060K.

[S2] Y. Cao, R. Yang, W Yang, Chinese Patent CN1389488 A, 08-01-2003.
